# Supplementary material for: Nucleotide asymmetry and flexible linker dynamics modulate drug efflux cycle of P-glycoprotein, A computational study
Source: Comput Struct Biotechnol J. 2025 Oct 31;27:4825–37. doi: 10.1016/j.csbj.2025.10.064 (PMC12636386; doi:10.1016/j.csbj.2025.10.064)
Supplement: Supplementary file 1 — Supplementary material [file mmc1.pdf]

Supporting Information for

Nucleotide Asymmetry and Flexible Linker Dynamics Modulate Drug Efflux Cycle of P-Glycoprotein, a Computational Study.

Sungho B. Han[1,2], Jim Warwicker[1], Hao Fan[2], Stephen M. Prince[1].

[1] School of Biological Sciences, Faculty of Biology, Medicine and Health, The University of Manchester, Oxford Rd, Manchester M13 9PL, United Kingdom.

[2] Bioinformatics Institute (BII), Agency for Science, Technology and Research (A*STAR), Singapore, 30 Biopolis Street #07-01 Matrix Singapore 138671.

This PDF file includes:

Figures S1 to S8
SI References

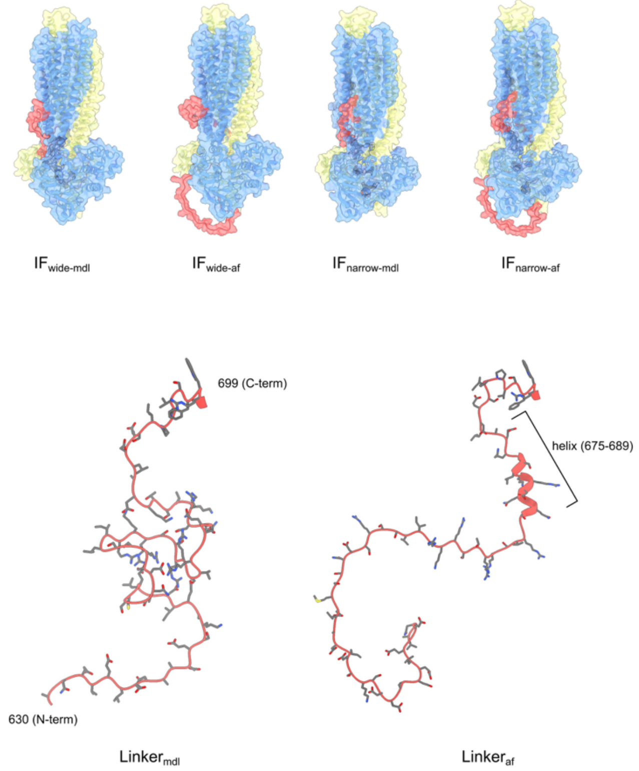

Fig. S1. Alternative surface view of the initial inward-facing human P-gp models and the cartoon representation of the two distinct linker structures used (linker-mdl and linker-af for linker configurations generated using Modeller and AlphaFold, respectively) (1, 2). Two halves of the transporters are colored in blue and yellow, respectively. The flexible linker connecting NBD1 and TMD2 is colored in red.

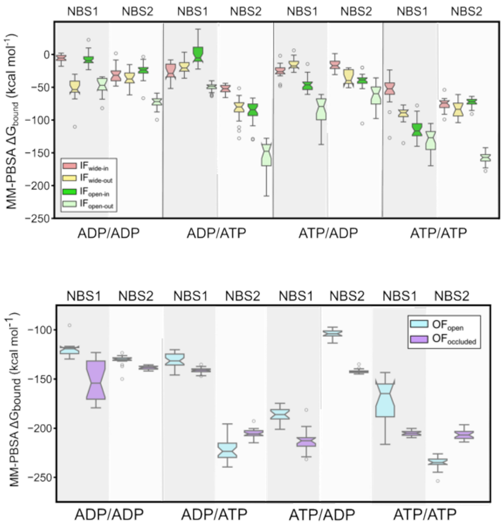

Fig. S2. Binding energies of nucleotides in P-gp calculated using MM-PBSA (3) in four nucleotide states. Top panel shows the energies from the inward-facing P-gp conformers. The bottom panel shows the energies from the outward-facing P-gp conformers.

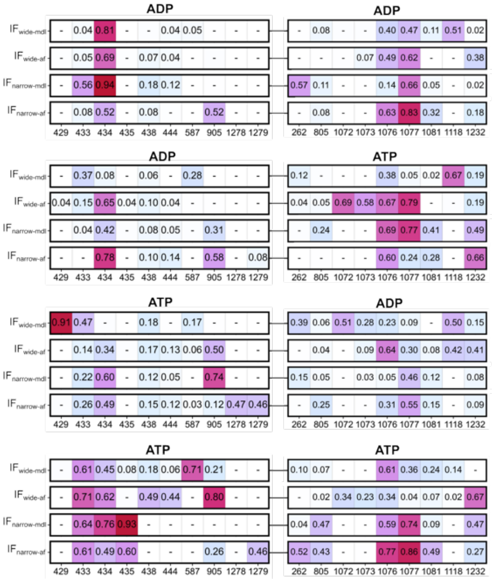

Fig. S3. Electrostatic interaction frequency between the bound nucleotide and nearby NBD residues in inward-facing P-gp conformers are shown as heatmap. The respective interaction frequencies are shown within the box along with the colors ranging from light blue to red. The interaction frequencies are clustered into four based on the four nucleotide states deployed in this study (ADP/ADP, ADP/ATP, ATP/ADP, ATP/ATP). The residue numbers of the NBS residues that interact with respective nucleotides are shown at the bottom of each heatmap cluster.

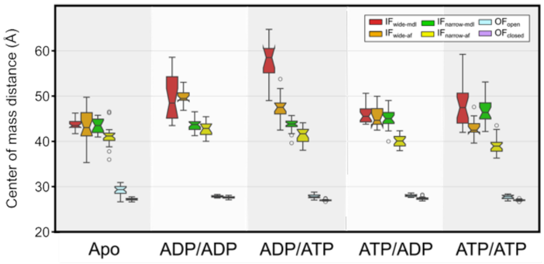

Fig. S4. Distribution of the center-of-mass distance between NBD1 and NBD2 in all P-gp MD simulations. Calculation was performed from the generated MD trajectories using CPPTRAJ (4).

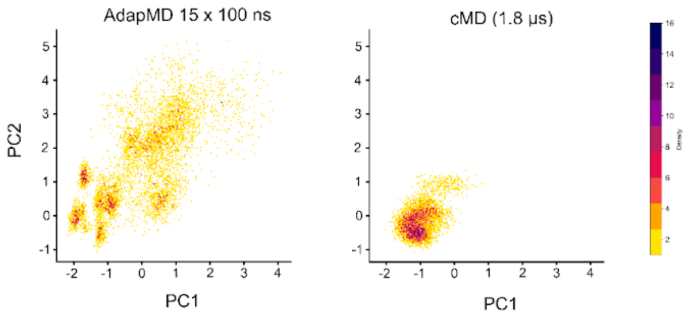

Fig. S5. PCA analysis of global P-gp conformation with standard single trajectory classical MD (cMD) simulation versus the current work's adaptive MD sampling approach (AdapMD). PC1/2 values are represented as a heatmap density using kernel density estimation. The distance between every fourth of 44-1272 residues in P-gp were used to featurize the trajectories. Fifteen 100 ns long trajectories that were extended based on multi-replica MD approach were used to construct the PCA heatmap for AdapMD. A single 1.8 µs long trajectory was used for constructing the PCA heatmap for cMD.

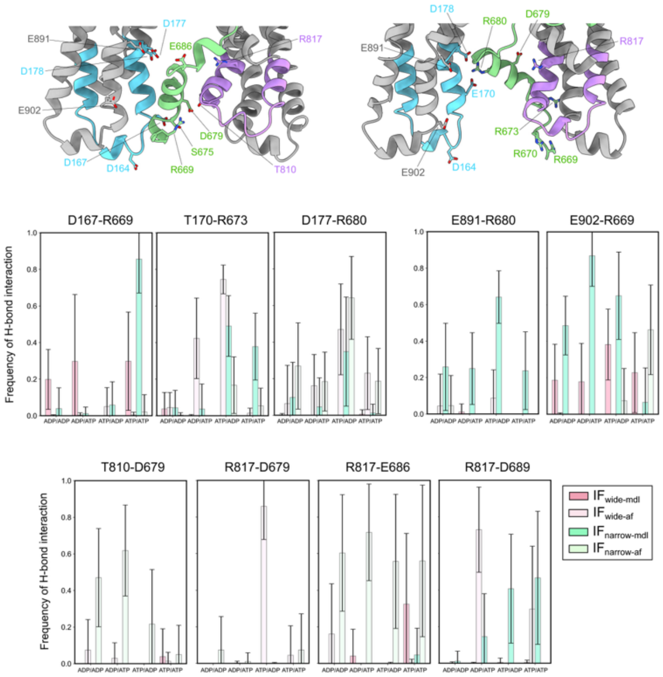

Fig. S6. The electrostatic interaction profile between the linker and NBD residues in IF P-gp conformers. The image at the top represents the intracellular segment of TMD in P-gp and the helical portion of the flexible linker. TMD1 segment is colored in grey/cyan, TMD2 segment is colored in grey/purple, and the linker is colored in green. The electrostatic interaction frequencies of the respective residue pairs are shown below.

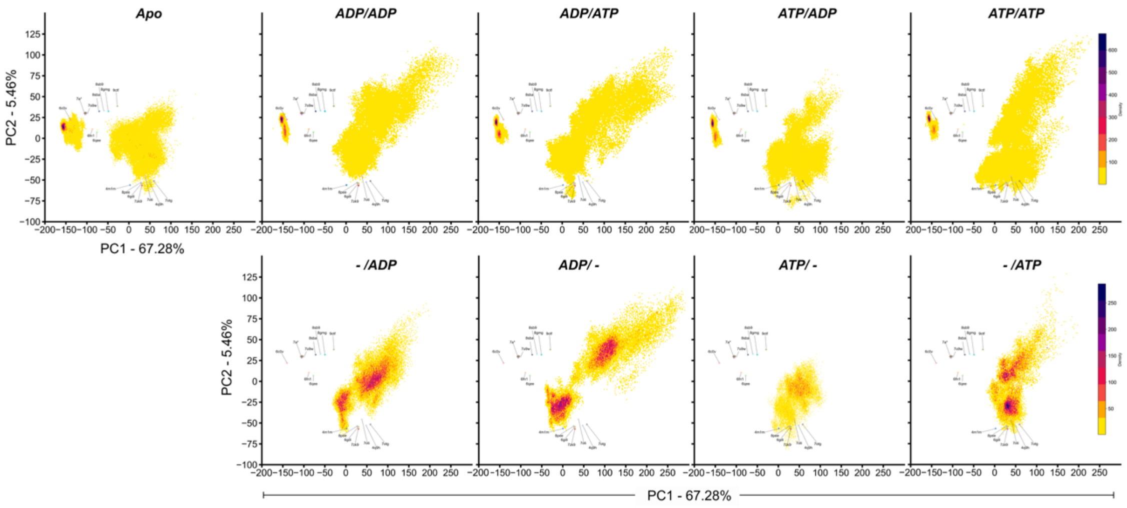

Fig. S7. PCA analysis of global P-gp conformation from MD simulation trajectories, each bound to different nucleotides. PC1/2 values are represented as a heatmap density using kernel density estimation. The distance between every third of residues 55-80,110-615, and 716-1256 in P-gp were used to featurize the trajectories (experimentally unresolved residues excluded). The known structures of P-gp were plotted along with the simulated data, labelled with the respective PDB access codes. PC1 and PC2 on axes are labelled with their variance contributions. 7a* refers to the cluster of points corresponding to PDB codes 7a6c, 7a6e, 7a69, 7a65, 7a6f, 9ctc and 6qex.

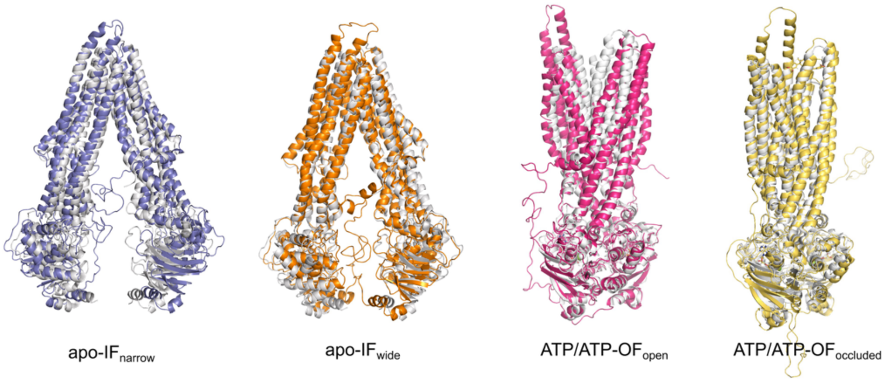

Fig. S8. Structural comparison of starting configurations of P-gp used in this study versus the recently deposited cryo-EM P-gp models (5). The cryo-EM structures are colored in white (PDB structures, 8gmg and 8sb8, were overlapped with IF and OF models, respectively). The RMSD of the residues 44-630,710-1272 between the cryo-EM models and the starting conformations of MD are labelled accordingly. These four conformers of P-gp each represent a key conformational state that likely occur across the ligand transport cycle.

SI References

1. N. Eswar, *et al.*, “Comparative Protein Structure Modeling Using MODELLER” in *Curr Protoc Protein Sci*, (2007), p. Unit\ 2.9.

2. J. Jumper, *et al.*, Highly Accurate Protein Structure Prediction with AlphaFold. *Nature* **596**, 583–589 (2021).

3. B. R. Miller, *et al.*, *MMPBSA.py*: An Efficient Program for End-State Free Energy Calculations. *J. Chem. Theory Comput.* **8**, 3314–3321 (2012).

4. D. R. Roe, T. E. Cheatham, PTRAJ and CPPTRAJ: Software for Processing and Analysis of Molecular Dynamics Trajectory Data. *J Chem Theory Comput* **9**, 3084–3095 (2013).

5. A. T. Culbertson, M. Liao, Cryo-EM of Human P-Glycoprotein Reveals an Intermediate Occluded Conformation During Active Drug Transport. *Nat Commun* **16**, 3619 (2025).
